# A Multifunctional Brain-Computer Interface Intended for Home Use: An Evaluation with Healthy Participants and Potential End Users with Dry and Gel-Based Electrodes

**DOI:** 10.3389/fnins.2017.00286

**Published:** 2017-05-22

**Authors:** Ivo Käthner, Sebastian Halder, Christoph Hintermüller, Arnau Espinosa, Christoph Guger, Felip Miralles, Eloisa Vargiu, Stefan Dauwalder, Xavier Rafael-Palou, Marc Solà, Jean M. Daly, Elaine Armstrong, Suzanne Martin, Andrea Kübler

**Affiliations:** ^1^Institute of Psychology, University of WürzburgWürzburg, Germany; ^2^g.tec Medical Engineering GmbHSchiedlberg, Austria; ^3^eHealth Unit, Eurecat - Technology Center of CataloniaBarcelona, Spain; ^4^The Cedar FoundationBelfast, UK

**Keywords:** brain-computer interface, EEG, practical electrodes, assistive technology, end-user evaluation

## Abstract

Current brain-computer interface (BCIs) software is often tailored to the needs of scientists and technicians and therefore complex to allow for versatile use. To facilitate home use of BCIs a multifunctional P300 BCI with a graphical user interface intended for non-expert set-up and control was designed and implemented. The system includes applications for spelling, web access, entertainment, artistic expression and environmental control. In addition to new software, it also includes new hardware for the recording of electroencephalogram (EEG) signals. The EEG system consists of a small and wireless amplifier attached to a cap that can be equipped with gel-based or dry contact electrodes. The system was systematically evaluated with a healthy sample, and targeted end users of BCI technology, i.e., people with a varying degree of motor impairment tested the BCI in a series of individual case studies. Usability was assessed in terms of effectiveness, efficiency and satisfaction. Feedback of users was gathered with structured questionnaires. Two groups of healthy participants completed an experimental protocol with the gel-based and the dry contact electrodes (*N* = 10 each). The results demonstrated that all healthy participants gained control over the system and achieved satisfactory to high accuracies with both gel-based and dry electrodes (average error rates of 6 and 13%). Average satisfaction ratings were high, but certain aspects of the system such as the wearing comfort of the dry electrodes and design of the cap, and speed (in both groups) were criticized by some participants. Six potential end users tested the system during supervised sessions. The achieved accuracies varied greatly from no control to high control with accuracies comparable to that of healthy volunteers. Satisfaction ratings of the two end-users that gained control of the system were lower as compared to healthy participants. The advantages and disadvantages of the BCI and its applications are discussed and suggestions are presented for improvements to pave the way for user friendly BCIs intended to be used as assistive technology by persons with severe paralysis.

## Introduction

Brain-computer interfaces (BCIs) based on event-related potentials (ERPs) are widely used in research settings (Kleih et al., 2011; Mak et al., 2011). Research confirms that the majority of healthy study participants were able to gain control over an ERP-BCI within the first session (Guger et al., 2009). Furthermore, users with degenerative neuromuscular disorders and a varying degree of paralysis were able to control an ERP-BCI spelling application (Kübler and Birbaumer, 2008; Nijboer et al., 2008; Sellers et al., 2010; Kaufmann et al., 2013a,b; Käthner et al., 2015a,b; McCane et al., 2015). In the classic ERP (P300) speller introduced by Farwell and Donchin (1988), rows and columns of a letter/symbol matrix are highlighted in random order. The participants are asked to focus on the symbol that they wish to select and to silently count, whenever it is highlighted. This paradigm is a variant of the oddball paradigm in which the attended rare target stimulus elicits specific ERPs of which the P3 and the N2 are the most prominent in an ERP-BCI (Kaufmann et al., 2011a). Due to the differences in the elicited ERP waveforms for the attended compared to the unattended stimuli, the target symbol can be identified at the intersection of the row and column that contain the target symbol.

Apart from its function as a muscle-independent communication aid, several other applications have been implemented. These include applications for artistic expression (*Brain Painting*), a web browser, a multimedia player, games intended for cognitive rehabilitation and switches for environmental control (Münßinger et al., 2010; Carabalona et al., 2012; Yu et al., 2012; Botrel et al., 2015; Halder et al., 2015; Holz et al., 2015a,b). The vast majority of studies with ERP-BCIs have been conducted with healthy participants in controlled laboratory environments (Kübler et al., 2013). These studies used soft- and hardware optimized for research purposes and required expert knowledge to set-up and configure the system. However, BCIs are intended to be used as assistive technology in end users' homes. Therefore, efforts are necessary to transfer BCIs from laboratory to home environments.

**Figure 1:**
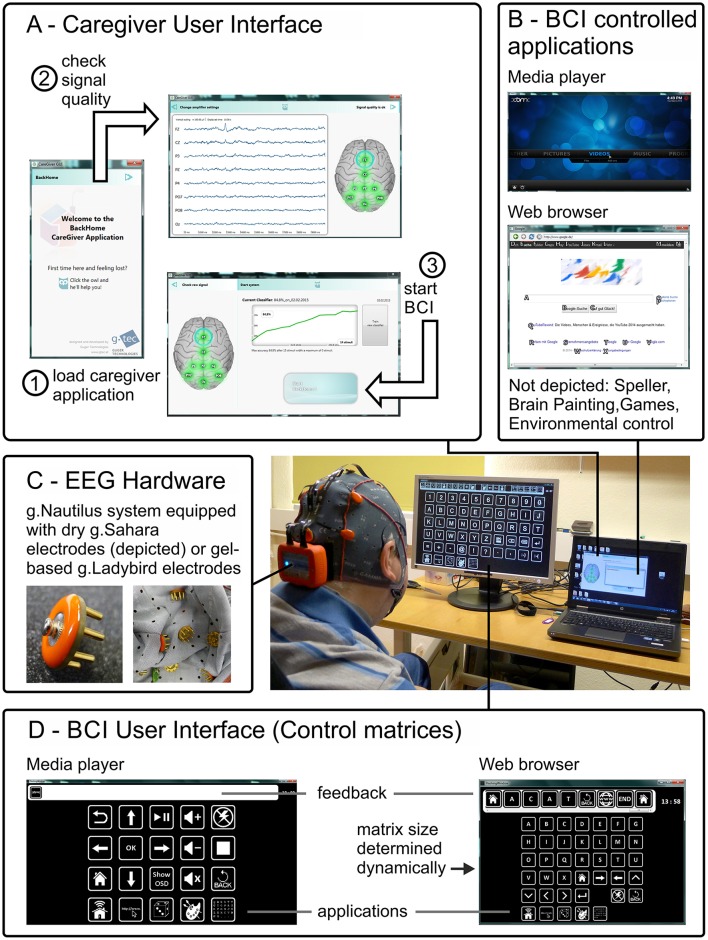
**Components of the BCI prototype developed within the project Backhome**. Caregivers and/or researchers are guided through the process necessary to start the BCI in a stepwise procedure with a graphical user interface **(A)**. The user is seated in front of the screens used to display the applications **(B)** and control matrices **(D)**. The EEG Hardware **(C)** consists of the g.Nautilus system that is either equipped with dry contact (depicted) or gel-based electrodes. The control matrices **(D)** contain a fixed number of elements except for the web browser, for which the number of elements is determined dynamically depending on the number of hyperlinks on the web page. For all other applications, the number of elements in the matrix depend on their functionality (e.g., the speller matrix contains more elements than the control matrix for the media player).

Within the European FP7 project *Backhome*^1^ we aimed at developing an ERP (P300)-BCI that is multifunctional and easy to use and configure. Therefore, we designed and evaluated prototypes within a user-centered process to continuously improve the soft- and hardware (Kübler et al., 2014). Previous hardware and software solutions were targeted to EEG experts. To facilitate home use, a number of improvements were made within *Backhome* to allow for a simple setup and at the same time versatile functionality, as outlined below. Evaluation results for a previous prototype have been published (Käthner et al., 2014; Daly et al., 2015a,b). The development efforts resulted in a final prototype that was first described by Miralles et al. (2015a). The system is outlined briefly in the following section and its main components are illustrated in Figure 1.

The hardware for the recording of EEG signals developed within *Backhome* consists of electrodes that are arranged in an electrode cap made of flexible fabric. These are connected to a small and lightweight, waterproof amplifier that is attached to the back of the cap and submits the EEG signals wirelessly to a computer (g.Nautilus). The system can be operated with active gel-based or dry contact (g.Sahara) electrodes.

To allow the caregivers to set-up the soft- and hardware without expert supervision, the final *Backhome* prototype, consists of a graphical user interface that guides the caregivers through the steps necessary to set-up the system and enable the BCI user to control the various applications afterwards (Miralles et al., 2015b). All the applications mentioned above (speller, web browser, multimedia player, Brain Painting, games for cognitive rehabilitation, switches for environmental control) were integrated into the prototype.

Miralles et al. (2015b) reported on the results of a 6-week home based evaluation with the final *Backhome* Prototype. Two users performed predefined tasks with the BCI supported by their caregivers, who had been trained on how to set up the system. Although both users expressed to be satisfied with the device, only 61 and 72% of the tasks could be completed respectively and technical difficulties proved to be challenging for the caregivers.

In the current study, for the first time, we investigated whether the final BCI prototype can be operated with satisfactory and similar accuracies with both the gel-based and dry electrodes by healthy participants. A second aim was to test the potential of the system for use in daily life of persons in need for muscle-independent communication and control. For this, persons with a varying degree of motor impairment tested the BCI during supervised sessions.

Therefore, a study in which two groups of healthy users had to complete an experimental protocol that consisted of performing tasks with the different applications (e.g., playing a video with the multimedia player, posting a message on facebook, turning on/off a light) was conducted. One group performed the tasks with gel-based electrodes and the other with dry electrodes.

Conventionally, wet electrodes are used in research settings, because they allow for high quality EEG recordings from the scalp. Commonly Ag-AgCl electrodes are mounted in a cap and a conductive gel is applied to ensure good contact between the electrode and the skin. The application of the gel results in considerable preparation time prior to recordings and to remove the gel after use, the hair needs to be washed which constitutes an issue in people with severe paralysis. Dry contact electrodes of various shapes and materials were proposed to simplify the set-up procedure and facilitate long-term recordings (see Liao et al., 2012b, for a review).

For dry electrodes, it is necessary to establish good contact with the skin to achieve satisfactory impedance levels and signal quality, while also ensuring good wearing comfort of the electrodes (Lopez-Gordo et al., 2014). The previously proposed sensors include various spring loaded metal pins (Liao et al., 2011; Lin et al., 2011; Toyama et al., 2012; Lo et al., 2016), electrically conductive foam (Lin et al., 2011; Liao et al., 2012a) silicon based pins (Yu et al., 2014), metal coated polymer bristles (Grozea et al., 2011) and micro-needles inserted beneath the skin (Griss et al., 2001; Carabalona et al., 2009)—see Lopez-Gordo et al. (2014) for an overview about EEG systems based on dry electrodes and their respective advantages and disadvantages. Nathan and Jafari (2014) investigated various configurations of metal pins as sensors and used the dry electrodes (g.Sahara) employed in our study as a benchmark, which yielded favorable results in terms of impedance, signal quality and robustness. In a feasibility study Guger et al. (2012) evaluated an ERP speller with the dry g.Sahara electrodes. With a fixed number of 15 sequences, all 23 participants were able to spell a five-letter word correctly. Within the framework of the *Backhome* project, Pinegger et al. (2016) evaluated the g.Sahara electrodes with an ERP spelling task, the multimedia player and web browser and adjusted the number of sequences for online use according to the results achieved during a classification run (100% + 2 sequences). With an average of 12.7 sequences, the 7 participants reached an average 87% correct for spelling 10 letters in the beginning of the experimental protocol and 70% in the end. The achieved accuracies for the multimedia player and web browser tasks (minimum of 10 selections required) were 87 and 64%, resulting in an overall accuracy of 77% (±11.8). The noise level of the dry electrodes was low (root mean square of 0.82 μV within the frequency range of 0.1–40 Hz) but higher as compared to gel-based electrodes (0.68 μV; Pinegger et al., 2016). Zander et al. (2011) compared the prototype of a three channel dry electrode system to conventional active gel-based electrodes regarding the performance in a variant of the oddball task. Although the average online accuracy of the 12 participants was slightly lower with the dry (72%) compared to the gel-based electrodes (78%) it was not statistically different.

To investigate the external validity, the final *Backhome* prototype was evaluated in our study by potential end users with a varying degree of motor impairment during supervised sessions in their home or caregiving environment. In these case studies, parameters were optimized to maximize performance with the system for each user. Following the user-centered design (UCD) performance of the BCI was evaluated and feedback of the users gathered to assess the usability of the system. Advantages and possible disadvantages of the prototype were revealed indicating the need for improvement in the future.

## Methods

We evaluated the final BCI prototype implemented within the project *Backhome*. It consists of newly developed hardware for data acquisition (g.Nautilus headset, g.tec, Austria) and software (Figure 1). For a thorough description of the individual elements of the system we refer to Hintermüller et al. (2015) and Miralles et al. (2015a).

### Healthy participants and data acquisition

Two groups of ten healthy participants each took part in the study. One group tested the system with gel-based (g.Ladybird; g.tec, Austria) electrodes (9 naïve to BCI, 6 female, mean age: 24.5 ± 3.4 years, range: 19–29 years; not age-matched with end users) and the other with the dry contact (g.Sahara) electrodes (all naïve to BCI, 9 female, mean age: 24.4 ± 2.7 years, range: 21–28 years; not age-matched with end users). Data collection with the gel-based electrodes started after the study with the dry electrodes had been finished. All participants were paid 8 Euro per hour for their participation. None of the participants reported a history of neurological or psychiatric illness. All participants signed informed consent prior to participation in the study, which was approved by the Ethical Review Board of the Institute of Psychology, University of Würzburg.

The data was acquired with a g.Nautilus headset from 8 electrodes (Fz, Cz, Pz, P3, P4, PO7, PO8, Oz) with a ground electrode positioned at FPz. The electrodes were fixed in a medium size cap made of flexible fabric. For the gel-based Ag/AgCl g.Ladybird electrodes the reference electrode was clipped to the right earlobe. For the dry gold coated g.Sahara electrodes a combination of the available short (7 mm) and long pins (16 mm) was used to ensure good contact with the scalp. The reference electrode was attached to the right mastoid and the ground electrode to the left mastoid (both using disposable Ag/AgCl electrodes).

The electrodes were connected to an amplifier attached to the back of the cap that transmitted the signals wirelessly to a base station (sampling rate 250 Hz). Data was recorded and the applications displayed with a Hewlett-Packard ProBook 6460b with a dual-core CPU, 4 GB of RAM and a 64-bit Windows 7. An external 22” LCD monitor (LG Flatron E2210) displayed the symbol matrices used to control the different applications, henceforth referred to as control matrices.

The number of elements in the control matrices was fixed for the different applications (see Figure 1) except the ones that controlled the web browser. The web browser is an improved version of the one described by Halder et al. (2015) that determines the number of elements in the control matrix dynamically depending on the number of hyperlinks displayed on the web page. Each hyperlink is overlaid with a letter or combination of two letters on the screen displaying the web browser. Compared to the previous version, these hints (letters) were more clearly silhouetted against the web page. The hints are displayed in the control matrix along with 8 icons for navigation, an icon for pause and one for back function. A maximum of 14 × 6 (84) elements can be displayed in the control matrix. In the main menu of the web browser (3 × 5 elements) up to 10 shortcuts to web pages can be defined (in the default mode, icons for facebook, google search, youtube, news page, weather forecast, and email are displayed).

At the bottom of each control matrix, 5 icons were displayed to allow switching between the applications (smart home control including multi media player, web browser, games intended for cognitive rehabilitation, Brain Painting, speller). The experimental protocol (described below) included tasks with all applications except the Brain Painting application, which has already been thoroughly evaluated by end users (Botrel et al., 2015; Holz et al., 2015a,b).

### Procedure for healthy participants

The external monitor displaying the control matrices was positioned in front of the participants, who were seated in a comfortable chair. Participants were asked to adjust the distance to the screen such that they were able to see properly all icons. The laptop displaying the applications was placed next to the monitor.

The caregiver interface was started to judge the signal quality and, if signal quality was sufficient, start the calibration run (Figure 1A). For caregivers, it depicts a simple color-coding system, where an electrode position marked red indicates that no signal is obtained and the electrode connection should be checked. Yellow indicates that the signal is outside an acceptable range (amplitude >100 μV) or artifacts are detected (variance >25 μV within a 1 s window) and contact to the skin should be improved until all electrodes are highlighted in green. The caregiver interface also depicts the electroencephalogram for each electrode. Prior to testing, the signal was visually inspected and assured that eye blinks were clearly visible and the alpha wave was apparent, when participants closed their eyes.

At first, each participant had to perform a calibration run. In this run, a 5 × 10 letter/symbol matrix was displayed on the monitor. Participants were instructed to focus on a given symbol and silently count whenever it was overlaid. During operation, the rows and columns were overlaid in random order with black and white photographs of Albert Einstein (Kaufmann et al., 2011b, 2013b). The pictures overlaying the icons were displayed for 120 ms and the interstimulus interval was set to 80 ms. Each row and column was overlaid 15 times. For the calibration, the BCI users had to focus consecutively on five symbols indicated by the system (copy spelling). At the end of the calibration run, the system automatically performed a classification using linear discriminant analysis and the performance estimation was displayed on the laptop screen (accuracy and number of sequences to achieve that accuracy).

In the following runs, the classification parameters were applied to allow the user to control the different applications. During these runs, the system selected the symbol determined by the classifier and additionally the selected icon was displayed in a line above the control matrix.

**Figure 2:**
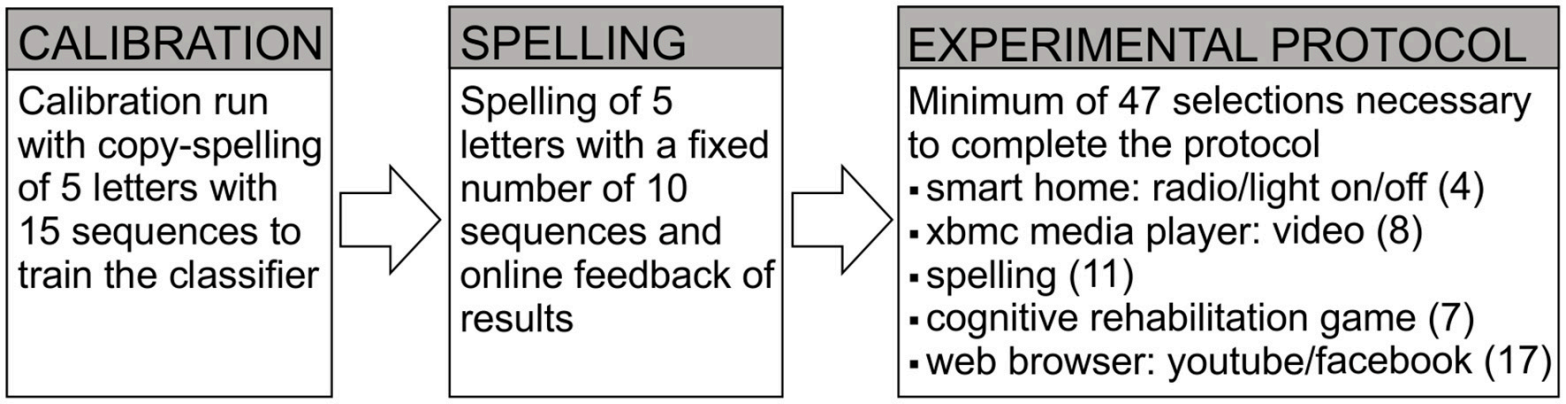
**Experimental Procedure for the testing of the prototoype**. For the experimental protocol, the number in parentheses behind the applications indicates the minimum number of selections necessary to complete the individual tasks. For the dry electrodes, the number of sequences was fixed to 10 during the experimental protocol. For the gel-based electrodes, the dynamic stopping method was activated.

In the first task, the investigator selected the spelling application and the user was asked to spell a 5-letter word (“Hallo”) with a 6 × 10 symbol matrix with the above described method; the number of sequences was set to 10 (each row and column was overlaid 10 times), prior to a selection (Figure 2; Guger et al., 2012).

After the copy spelling run, a dynamic stopping method was activated (Schreuder et al., 2013). Henceforth, a selection was made as soon as a certain probability threshold was reached to increase the selection speed. If the threshold was not reached after a maximum number of ten sequences, the system refrained from a selection to minimize the number of false positive selections and continued flashing. In the following, we will refer to this case as “suppressed selection.” As determined in a pre-study, the dynamic stopping method did not work properly with the dry contact (g.Sahara) electrodes. In the pre-study with the dry electrodes, 10 healthy participants (7 female, mean age 24.2 ± 3.4) performed the same experimental protocol as in the current study, but with the dynamic stopping method activated. The maximum number of sequences for online use was set to 10. If the probability threshold for a selection was not reached after 10 sequences, the system kept on flashing and the selection was marked as “suppressed.” In the pre-study, 30.6% of selections were wrong and in 45.5% of cases, the selections were suppressed.

For these reasons the dynamic stopping method was turned off in the current study for the second experimental group that tested the dry electrodes and a selection was always executed after ten sequences (each row and column flashed 10 times).

To determine the accuracy of the system, the users were instructed to select predetermined icons with different control matrices. In order to complete the experimental protocol a minimum of 47 selections had to be made. The experimental protocol consisted of turning on and off two remote control sockets, which turned a light/radio on/off, playing a video with the XBMC media player (Halder et al., 2015), writing “DREAMTEAM ☺” with the spelling application, completing the first level of the cognitive rehabilitation task (Vargiu et al., 2014), playing a video on YouTube, writing the word “QUIZMASTER” as status text on Facebook and selecting icons to switch between the applications. Figure 2 depicts schematically the experimental procedure.

If a wrong icon was selected it was marked as a wrong selection. If the dynamic stopping method did not make a selection after 10 sequences, flashing continued and we marked this case as “suppressed selection”. In both cases, the user was asked to select the same icon. For each icon the user had a maximum of three attempts. If the user was unable to select an icon within three attempts, the icon was selected manually by the investigator and the icon marked as “unable to select.” The control matrices differed for each application. Selections had to be made with a total of eight different control matrices. The time needed to complete the protocol was noted along with the respective number of selections (e.g., if two selections were suppressed and one selection was wrong, 50 instead of the minimum of 47 selections were required to complete the protocol.

We calculated the average percentage of icons that the users were able to select within three attempts and the average percentage of false selections. Furthermore, we calculated the average percentage of suppressed selections to assess the performance of the dynamic stopping method during testing with the gel-based electrodes. To calculate the percentage of icons the participants were able to select within three attempts the following formula was used for each participant:

$$\begin{array}{l} 100-\left(\frac{total\;number\;of\;icons\;unable\;to\;select}{47\;}*100\right) \end{array}$$

To calculate the percentage of wrong/suppressed selections the following formula was applied:

$$\begin{array}{l} \frac{total\;number\;ofwrong|suppressed\;icons}{total\;number\;of\;selections\;}*100 \end{array}$$

### Usability assessment

Effectiveness, efficiency and satisfaction are the three aspects of usability according to the definition by the ISO (ISO 9241–210, 2008). Different measures were previously proposed to operationalize these aspects for BCI controlled applications to allow for a user-centered BCI development and evaluation (Zickler et al., 2011; Kübler et al., 2014). These measures provide a standard for usability assessment in the BCI context and can be adapted and supplemented according to the applications of interest.

To assess how accurate and complete (effective) the users accomplished the tasks, we calculated the accuracy as the percentage of correct responses, the percentage of icons users were able to select within three attempts and the number of suppressed selections as described above.

Efficiency relates the costs invested by the user to effectiveness. As a measure of efficiency, the subjective workload was assessed with an electronic version of the NASA Task Load Index (NASA-TLX; Hart and Staveland, 1988). Each of the six factors (mental, physical and temporal demands, effort, own performance, and frustration) had to be rated on a 20 point Likert-type scale (0–100). In a second step, the participants indicated, in a pair-wise comparison of all six factors, which contributes more to the workload. Weights are assigned to each factor according to this procedure by dividing the number of times a factor is chosen as more relevant and dividing this value by 15 (the total number of paired comparisons). This weight is then multiplied with the respective dimension score. The summation of all weighted scores yields a total score ranging from 0 to 100, where a high score indicates high workload.

To assess satisfaction with the BCI, the participants were asked to mark their overall satisfaction with the BCI on a visual analog scale (VAS) ranging from 0 (= not satisfied at all) to 10 (= very satisfied) after completing the experimental protocol.

Further, satisfaction was assessed with an extended version of the Quebec User Evaluation of Satisfaction with assistive Technology (eQUEST 2.0; (Zickler et al., 2011) and a usability questionnaire concerning the system design. The original QUEST 2.0 (Demers et al., 2002) consists of 12 items that have to be rated on a 5 point scale to indicate the level of satisfaction with different aspects of the assistive technology (1 = not satisfied at all, 2 = not very satisfied, 3 = more or less satisfied, 4 = quite satisfied, 5 = very satisfied). For the BCI specific eQUEST 2.0 (Zickler et al., 2011) eight items of the original questionnaire were adopted [dimensions, weight, adjustment, safety, comfort, ease of use, effectiveness, professional services (information/instructions)] and 4 items added to assess also reliability, speed, learnability and aesthetic design of the BCI prototype. To calculate a total score of the eQUEST 2.0 only the average of the 8 original items was calculated to ensure validity of the questionnaire and the average for the BCI specific items is reported separately. Whenever the users indicated that they were not very satisfied with the system they were asked to comment. At the end of the questionnaire, the users were asked to indicate the three most important items.

To gather further feedback from the users, we specifically asked them to name a positive and a negative experience and suggestions on how to improve the system and a set of closed-ended questions: Did you feel in control while using the system? Did you get useful feedback from the system while you were using it? Did you find the system intuitive? Did you like the colors used on the screen? Did you like the pictures/icons used on the screen?

### Participants with motor impairments

All six participants gave informed consent using their standard communication channel prior to participation in the study, which was carried out in accordance with the recommendations of the Ethical Review Board of the Institute of Psychology, University of Würzburg. Participants 1, 2, and 3 gave written and informed consent and on behalf of participants 4, 5, and 6 their respective legal guardians gave written and informed consent in accordance with the Declaration of Helsinki. The protocol was approved by the Ethical Review Board of the Institute of Psychology, University of Würzburg.

An overview of the potential end users, who participated in the evaluation is listed in Table 1.

**Table 1:** **Information on health status of end users**.

**End user**	**Sex**	**Age**	**Diagnosis**	**Degree of motor-impairment**	**Verbal speech**	**Artificial ventilation**
01	Female	59	Cerebral palsy	Tetraparesis, residual control over right arm and hand	Slurred	No
02	Male	34	Cerebral palsy	Spastic tetraparesis	Intact	No
03	Male	51	Stroke	Hemiparesis	None	No
04	Male	26	Lipid storage myopathy	Tetraparesis, only residual motor control over two fingers and eyes	None	Yes
05	Male	52	Spinal cord injury	Tetraplegia	Intact	No
06	Female	62	ALS	Locked-in state	None	Yes

The aim was to apply the same experimental protocol as in healthy subjects to assess the performance of the system with potential end users. However, due to individual differences in capabilities, the experimental protocol had to be adjusted to each participant and parameters had to be changed in an iterative process between the end-users and experimenters to optimize the performance of the system. For these reasons, the performance and usability assessments of the healthy participants and end users are not directly comparable and the findings with respect to end-users should be considered as stemming from individual case studies. The possible adjustments with the prototype will be described in the following paragraph and changes to the protocol will be described for each user in the subsequent sections.

For users with a restricted field of view, both the applications and the control matrices can be displayed on a single screen (split screen mode) and/or the window size of the control matrices can be adjusted. Other parameters that can be modified were the flashing stimuli (e.g., pictures of famous faces or a black and white picture of Albert Einstein) and the timing parameters for the flashing and the number of (maximum) stimuli prior to a selection.

In general, the adjustment process began with fitting the electrode cap and checking the signal quality as described in Section Procedure for Healthy Participants and illustrated in Figure 1A. Afterwards the classifier was trained (see Section Procedure for Healthy Participants and Figure 2). The estimated classification accuracy and optimal number of sequences to reach this accuracy were displayed automatically by the system after the classifier training ended. The determined accuracies and signal quality served as decision base, whether online spelling could be attempted. If online spelling yielded satisfactory accuracies (≥70% correct), the participants were asked to complete the tasks of the experimental protocol (see Figure 2). If signal quality was insufficient, we tried to improve the signal quality using the equipment available at the time of testing and repeated the classification run. The adjustment procedure could differ due to differences in available equipment, differences in the capabilities of the end-user and time constraints. The minimal goal was to allow participants to gain control over the spelling application and parameters mentioned above (window size, timing parameters, number of sequences, flashing symbol) were changed in an iterative procedure.

Tests with end users 1, 2, and 3 were conducted at an information center for Augmentative and Alternative Communication. Tests with end users 4, 5, and 6 were conducted at their respective homes during multiple sessions. Due to their severe motor impairments and necessary individual adjustments of the experimental protocol, they are described individually in the following.

#### End users 1–3

End users 1–3 (see Table 1) were sitting in their wheelchairs in front of the computer screens that displayed the control matrices (see Figure 1) during testing in single sessions. End users 1–3 were asked to test the g.Nautilus system with the dry electrodes (short pins) and a medium size cap. All others were offered to test both the dry (with long and short pin sizes) and the gel-based g.Nautlilus system. User 3 performed the experimental protocol with 12 instead of 10 sequences and started with the spelling task defined within the protocol followed by all other tasks. He did not wish to answer the NASA-TLX due to time restrictions.

#### End user 4

The participant was 26 years old and had been diagnosed with lipid storage myopathy (LSM) at the age of 2.5 years. This rare disorder, lead to a progressive loss of muscle control. At the time of the study, the participant was completely paralyzed except for residual control over two fingers of his right hand. With tiny movements of these fingers he could use a touchpad to control a computer program to write text. This method (dasher; Wills and MacKay, 2006) was also the one we used to communicate with him during testing. His vision was intact, but his field of view restricted to the size of his 22” computer monitor. Therefore, only his monitor was used to display the control matrices and/or applications. Due to ptosis his eyes had to be kept open with an aid. He had lost his hearing, therefore communication was only possible through his computer. Prior to the study he had tried to achieve control over an eye tracking system, but the attempts were unsuccessful.

**Figure 3:**
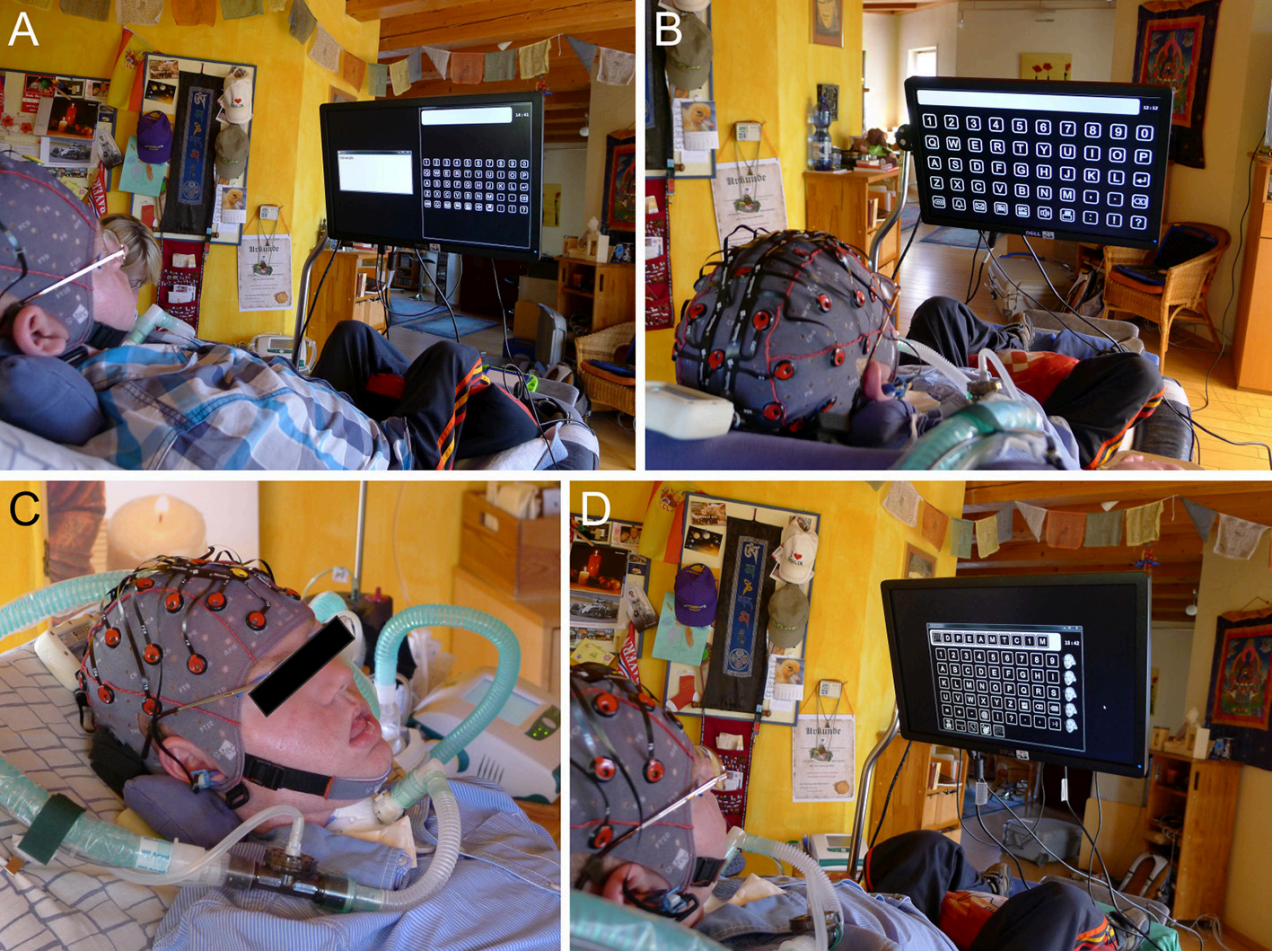
**End user 4 during testing of the prototype**. In **(A)** the split screen mode is shown. In **(B)** the spelling matrix is displayed in full screen mode. The selected symbols were displayed in the line above the matrix such that the user received feedback although the corresponding application window for spelling was not inside his field of view. **(C)** depicts a close-up of the user and the electrode cap and in **(D)** the window size of the spelling matrix is adjusted to fit the application window size of his conventional software (dasher) used for communication. End user 4 gained only rudimentary control over the spelling application and did not test the other applications. Pictures are published with consent from the participant and his legal representative.

During testing he was lying in a modified armchair and the 22” computer monitor was positioned in front of him with a flexible metal holder (see Figure 3).

He tested the system on three consecutive days. On the first day he tested the g.Nautilus with the dry g.Sahara electrodes and the system was displayed in the split screen mode (see Figure 3A). On the second day, he tested the system equipped with the gel-based electrodes first in the split screen and subsequently in the full screen mode (see Figure 3B). Using the full screen mode, we first tested the standard timing parameters (120/80 ms) and subsequently changed it to 200 ms/200 ms to decrease the stimulation frequency. Further, to ensure that all icons could be equally well recognized, we reduced the size of the window (see Figure 3D) to match the size used during his conventional communication (with the dasher program) and performed a run with the standard timing parameters. On day 3, we tested the system again with the same parameters as on day 2 (120/80 ms and window size adjusted).

#### End user 5

The participant was a 52-year-old male, who had a spinal cord injury 4 years and 8 month prior to the tests. He was tetraplegic and only able to move his head and neck. In daily life, he used an eye tracker to operate his computer.

**Figure 4:**
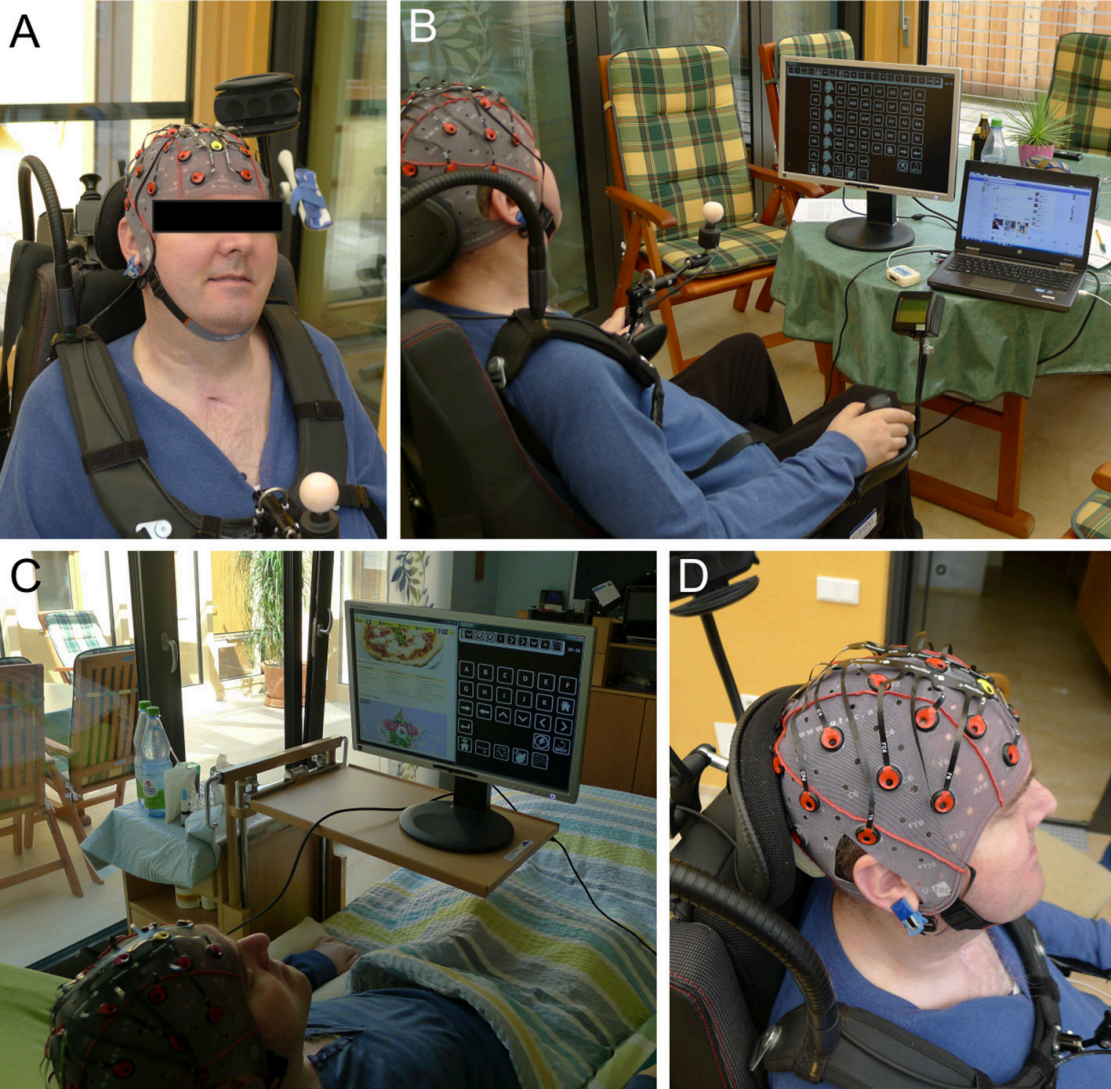
**End user 5 during testing of the prototype**. **(A)** depicts the user wearing a medium sized electrode cap with the gel-based electrodes. **(B)** shows him in his wheelchair during testing on day 1. **(C)** is a picture taken on day 2 of testing and **(D)** a close up of the electrode cap with the reference electrode attached to the right earlobe. End user 5 gained control over the system. Pictures are published with consent from the participant and his legal representative.

We tested the g.Nautilus equipped with the gel-based electrodes on two separate days. In the first session, the participant sat in his wheelchair in front of the computer screens used to display the applications and matrices. In the second session, the participant was lying in his bed and the system was displayed in the split-screen mode on a 22” monitor positioned such that the screen was within his field of view (see Figure 4). We then instructed his wife on how to set-up the system. She put on the electrode cap and also started the software by herself. We first asked him to spell a five-letter word (“HALLO”), afterwards he could explore the system by himself, while we explained the functions to him.

Due to a broken lead, no signal could be recorded from electrode PO8.

#### End user 6

**Figure 5:**
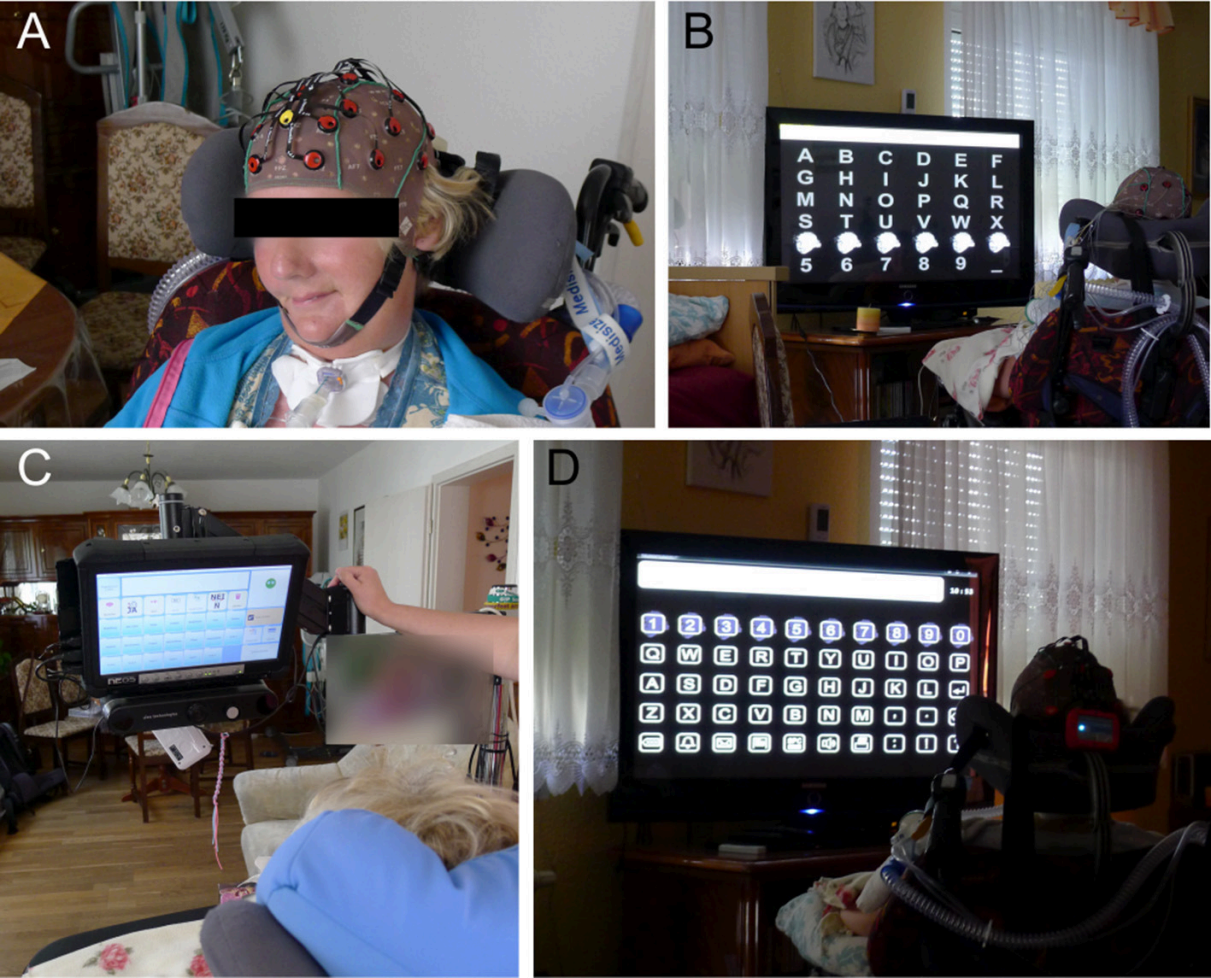
**End user 6 during testing of the prototype**. The end user wearing the electrode cap with the g.Ladybird electrodes **(A)**. **(B)** shows the end user seated in her wheelchair in front of the TV used to display the BCI2000 control matrix for spelling. The eye tracking system **(C)**. The calibration matrix displayed on the TV screen **(D)**. Pictures are published with consent from the participant and her legal representative.

The participant was a 62-year-old woman and had been diagnosed with amyotrophic lateral sclerosis 6 years prior to the study. She was artificially ventilated and fed and had only residual control of her eye movements. Binary communication was possible employing slow horizontal eye movement. Blinking was not possible. She had previously used an eye tracking system for communication (see Figure 5), but had only limited control of it during the last 4 weeks prior to testing due to a decline in her ability to control eye movements. Therefore, she only used it sporadically (about once per week) for short periods of time (about 20 min) according to her caregivers.

She participated in sessions on three consecutive days. On day 1, we first tested a medium size cap equipped with gel-based electrodes. The participant was sitting in her wheelchair with an external 22” monitor positioned in front of her. Secondly, we tested a smaller electrode cap equipped with the dry electrodes (long pins). Afterwards we switched back to the gel-based electrodes (small cap size) and displayed the calibration matrix on a large screen TV in front of her (see Figure 5). On day 2, we used the BCI2000 software framework to display a 6 × 6 letter matrix on her large screen TV (see Figure 5). EEG data was recorded with 8 gel-based g.Gamma electrodes mounted in a small cap and amplified with a g.USBamp. The same stimulation image was used as for testing with the *Backhome* prototype and timing parameters were identical. The participant performed a total of 7 runs, in which she had to select 5 target letters from the matrix respectively. During the stimulation, each row and column was flashed t10 times per letter selection. On day 3, we tested the *Backhome* prototype with the dry electrodes (small cap/long pins). The calibration matrix was displayed on the large screen TV. Afterwards we asked her and her caregiver to demonstrate the eye tracking system. It was positioned in front of her such that her eyes could be detected by the system (see Figure 5). At the end of testing we asked her a set of *ad-hoc* questions that she could answer with the help of her eye tracking system.

#### Caregivers

For end users 4–6 the relatives and or professional caretakers were present during the testing. Therefore, we included these stakeholders in the evaluation since we were also interested in their feedback about the ease of use of the caregiver interface and the set up procedure. If end users were able to control the BCI and caregivers agreed, we let them conduct the set up by themselves in a supervised session after we had explained the functionality of the soft- and hardware to them. To gather their feedback, we asked them to indicate how difficult/easy the setup up was with a VAS ranging from 0 (= very difficult) to 10 (= easy) and posed a range of questions about the usability of the system in general from the caregivers point of view and about the caregiver user interface in particular.

## Results

Results are first presented for the healthy study participants followed by the results of the end users in Section End Users.

### Healthy participants

#### Effectiveness

**Figure 6:**
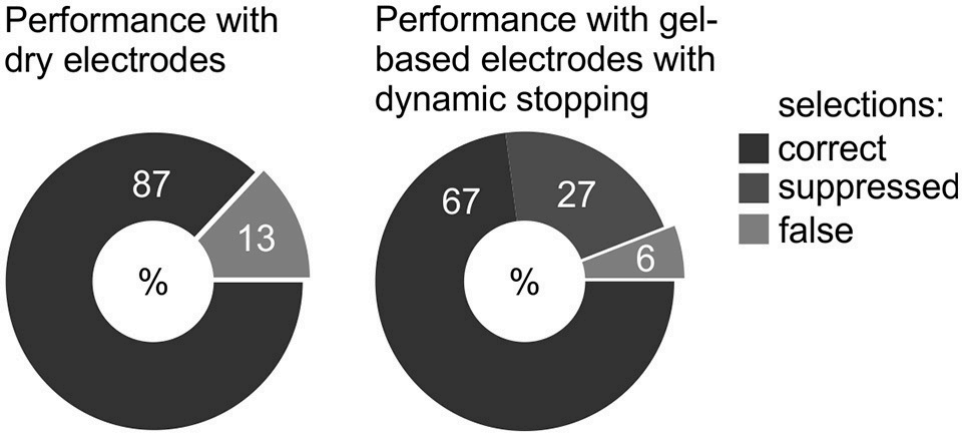
**Average accuracies of healthy participants during the experimental protocol with the gel-based and dry electrodes**. For the group with gel-based electrodes, the dynamic stopping method was activated, whereas for participants with the dry electrodes, the number of sequences for a selection was fixed to 10.

In the run in which participants spelled a five-letter word with a fixed number of 10 sequences, all subjects reached a spelling accuracy of 100% with both gel-based and dry electrodes. An overview about the participants' performance with gel-based and dry electrodes during completion of the tasks of the experimental protocol is depicted in Figure 6.

With the gel-based electrodes and the dynamic stopping method activated, 90% of icons could be selected, whereas with the dry electrodes significantly more icons (99%) could be selected within three attempts per icon (Mann-Whitney U-test. *U* = 14, *p* = 0.005).

Table 2 lists the performance for each healthy participant. Participants needed on average 24.1 ± 8.2 min to complete the experimental protocol with the gel-based electrodes and 28.8 ± 8.0 min with the dry electrodes (Mann-Whitney U-test. *U* = 47, *p* = 0.605; *N* = 9). These times do not include time for setup and the spelling task with a fixed number of 10 sequences. The selections per minute (and correct selections/min) for completing the protocol with the *Backhome* prototype can be roughly estimated from Table 2. The healthy users had an average of 2.8 selections per minute (1.8 correct selections/min) with the gel-based electrodes and 1.9 selections/minute (1.6 correct selections/min) with the dry electrodes.

**Table 2:** **Perfomance for individual participants for completing the experimental protocol with the dry or gel-based electrodes**.

	**Selections and time required to complete the tasks of the protocol**								
	**Healthy (Gel)**					**Healthy (Dry)**			
**Participant**	**Total**	**Suppressed**	**Wrong**	**Icons unable to select within three attempts**	**Time to complete the protocol (in minutes)**	**Total**	**Wrong**	**Icons unable to select within three attempts**	**Time to complete the protocol (in minutes)**
1	70	21	5	3	28	56	9	0	30
2	83	44	2	10	34	78	33	2	43
3	55	3	5	0	16	48	1	0	n/a
4	59	14	1	2	19	50	3	0	25
5	59	9	6	3	n/a	53	6	0	26
6	50	4	0	1	13	48	1	0	25
7	81	37	7	10	32	61	15	1	30
8	56	8	2	0	15	50	3	0	26
9	79	45	0	13	30	48	1	0	24
10	72	16	12	3	30	57	10	0	30
Mean	66.4 (±12)	20.1 (±16.2)	4 (±3.8)	4.5 (±4.7)	24.1 (±8.2)	54.9 (±9.3)	8.2 (±9.9)	0.3 (±0.7)	28.8 (±5.8)

The estimated number of sequences to obtain 100% accuracy in the calibration run was lower for the gel-based electrodes (3.9 ± 1.5) as compared to the dry electrodes (5.4 ± 2.3) albeit this difference did not reach statistical significance (Mann-Whitney U-test, *U* = 29.5, *p* = 0.112).

**Figure 7:**
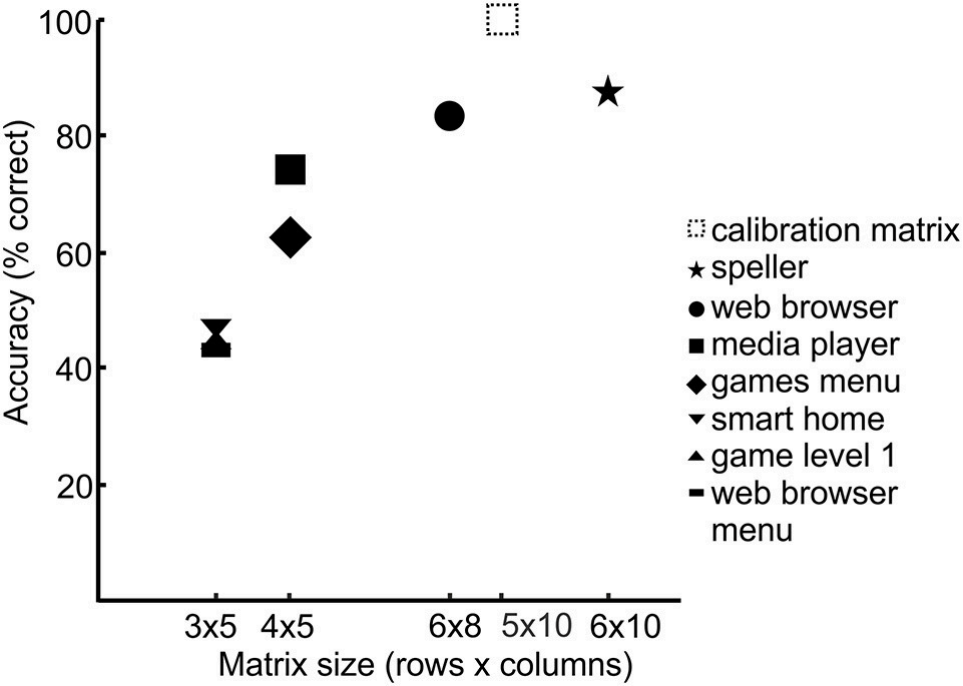
**Performance with the gel-based electrodes as a function of matrix size**.

It is apparent from Figure 6 and Table 2 that high a number of selections was suppressed with the gel-based electrodes and the dynamic stopping method activated. In the prototype, the size of the displayed icons was adjusted according to the size of the matrix, i.e., the less elements were in a matrix, the larger the size of the displayed icons (and the larger the difference in size as compared to the icons used for the calibration run). To explore whether there was a dependence of performance on the size of the control matrices and consequently of the size of the symbols we plotted Figure 7. For this, the average accuracies achieved with the different control matrices were calculated for the group that tested the gel-based electrodes.

#### Efficiency and satisfaction

The results of the eQuest2.0 are listed in Table 3, the overall satisfaction ratings with the VAS and the answers to the custom questionnaire on system design in Table 4 and the NASA-TLX scores are presented in Table 5. In the following we will complement and highlight some of the findings listed in these tables.

**Table 3:** **Satisfaction ratings assessed with the extended QUEST 2.0 and user comments**.

**Items**	**Healthy (Gel)**	**Healthy (Dry)**	**User 3 (Dry)**	**User 5 (Gel)**	**User comments**
**EXTENDED QUEST 2.0 (RATINGS FROM 1 TO 5)**					
Dimensions	4.4	4.5	3	3	Healthy (Gel): “smaller is always possible,” “a bit too tight around the chin,” “adjustment to different head shapes could be improved,” “ear-clip annoying in the long run” | End user 5: “cap feels very tight after wearing it for some time” | Healthy (Dry): “noticeable from the corner of one's eyes,” “small pressure, otherwise hardly noticeable”
Weight	4.7	4.3	4	4	Healthy (Gel): “unusual feeling that one will probably get used to” | Healthy (Dry): “somewhat heavy,” “very pleasant, light,” “one feels the cap in the beginning, but forgets about it after a few minutes of wearing it,” “after some time the electrodes press, the amp gets too heavy and pulls backwards”
Adjustment	4	4.4	2	3	Healthy (Gel): “unfortunately adding gel necessary,” “signal wasn't very good in the beginning” | End user 5: “pretty easy to set up the electrode system and adjust the cap” | Healthy (Dry): “slightly time-comsuming (for long hair),” “easy adjustment to different head shapes, easier compared to other EEG systems,” “easy readjustment,” “expertise required,” “help of participant required,” “required several attempts”
Safety	5	5	3	4	
Comfort	4.4	3.9	2	4	Healthy (Gel): “cap could be annoying in the long run,” “the cap is somewhat constricting,” “very comfortable, but long usage is strenuous for the eyes,” “usefulness high, but cap very conspicuous in public,” “good for home use, but not in public, “partly uncomfortable due to cap” | End User 5: “sweating, below the cap—it could be more breathable” | Healthy (Dry): “electrodes press slightly, but almost like a head-massage :); pressure on chin [through strap],” “the felt-pressure increases over time,” “not comfortable in the beginning, but hardly noticeable in the end,” “electrodes painful after some time,” “one feels cramped,” “small pressure,” “like a helmet,” “eye-catching in public, therefore not completely perfect”
Ease of use	4.4	4.5	2	2	Healtyh (Gel): “one has to practice,” ”varying symbol size impedes recognition” | End user 5: “eye tracker better” | Healthy (Dry): “long time until command executed,” “one can easily make mistakes if concentration is not high enough,” “strenuous for eyes, otherwise easy to control,” “would be optimal in combination with text auto completion software”
Effectiveness	4.5	4.3	2	2	Healthy (Gel): “selections take long sometimes,” “several small mistakes that one can undo easily,“ “time consuming,” “works well, but takes a bit long” | Healthy (Dry): “from time to time (very rarely) errors occurred,” “one needs a lot of patience and practice,” “a matter of practice,” “might not work for complex web pages, if a lot of scrolling is required”
Prof. Services (information/intsructions)	4.8	5	4	4	
QUEST 2.0 total score	4.5	4.5	2.8	3.3	
Reliability	4.5	4.5	2	5	Healthy (Gel): “more reliable than expected,” “did not recognize everything,” “reacts often different” | Healthy (Dry): “selected wrong icons sometimes,” “if own movements are too strong, position of electrodes can shift,” “sometimes the software failed,” “took some time in the beginning”
Speed	3.7	4.1	2	1	Healthy (Gel): “partly very slow,” “would need a long time to write text—too slow,” “for text quicker selections desireable, e.g., auto-completion,” “takes longer than using mouse/keyboard” | End user 5: “pretty slow compared to eye tracker” | Healthy (Dry): “takes a bit long,” “for a healthy study participant it is slow, but for a patient it could be a success,” “could be quicker (2),” “selections take too long”
Learnability	4.8	4.9	3	3	Healthy (Gel): “big effort necessary”
Aesthetic design	3.7	3.8	1	1	Healthy (Gel): “All in all acceptable,” “not really pretty, but that is not important [cap],” “not very fashionable but functional with a futuristic design [cap],” “too big, chunky [amp],” “a brick [amp],” “an EEG cap will probably never look pretty,” “very basic, but works flawless [software]” | Healthy (Dry): “design could be less conspicuous (2),” “conspicuous, but ok,” “big, conspicuous,” “100% eye-catching, in public all eyes would be on it,” “caps never look well,” “functionality is more important,” “slightly too big [amp],” “very basic design [software]”
Added items total score	4.2	4.3	2	2.5	

*QUEST2.0 = Quebec User Evaluation of Satisfaction with assistive Technology: 1 = not satisfied at all, 2 = not very satisfied, 3 = more or less satisfied, 4 = quite satisfied, 5 = very satisfied*.

**Table 4:** **Overall satisfaction ratings expressed with a visual analog scale (VAS) and answers to the custom usability questionnaire (system design)**.

	**Healthy (Gel)**	**Healthy (Dry)**	**User 3 (Dry)**	**User 5 (Gel)**
	**Visual analog scale (VAS) overall device satisfaction (0 = not satisfied at all, 10 = extremely satisfied)**			
	**7.5**	**7.8**	**3.8**	**4.5**
**CUSTOM USABILITY QUESTIONNAIRE (SYSTEM DESIGN)**				
Did you feel in control, while using the system?	Yes (*N* = 9)	Yes (*N* = 10)	Yes	Yes
Would you describe the system as intuitive?	Yes (*N* = 9)	Yes (*N* = 9)	Yes	No (regarding the functionality of some icons), otherwise yes
Operating the interface was…	Easy (4), ok (5), partly difficult (1)	Easy (6), ok (4)	Ok	Ok
Did you like the symbols/icons of the interface?	Yes (*N* = 7)	Yes (*N* = 7)	No	Yes
Did you like the colors of the interface?	Yes (*N* = 6)	Yes (*N* = 7)	n/a	Yes
Positive experience	“Success if attentive,” “spelling works quite well,” “one can reliably reach the overall goal,” “many senses of achievements,” “I would have never thought that something like this was possible,” “that the system recognizes the fixated icons and to control the program,” “I could write,” “positive how easy commands could be executed with the system,” “much was recognized correctly,” “controlling the interface”	“Control with eyes,” “weight of the cap,” “exciting to give commands basically just using the brain,” “control via eye gaze,” fascinating how well it worked, and I am happy if persons with paralysis could gain quality of life using this technology,” “writing with eyes,” “being able to test the usefulnes of this technology,” “control over systems without using hands possible,” “good cause, interesting, was fun,” “worked well,” “to control things just using gaze and attention”	“Should be 100% accuracy”	“Helping others by testing it”
Negative experience	“Tiring, because of repetitive flashing, constricting cap,” “controlling menu difficult,” “long time needed for selections,” “frustrating if wrong symbol gets selected,” “very tiring,” “the gel in my hairs,” “often it [the intended symbol] was not recognized correctly,” “did not work 100%,” “some symbols were not recognized,” “wrong selections”	“If wrong items are selected,” “I got tired quickly,” “takes a long time,” “attention difficulties,” “three wrong selections in a row,” “eyes ache slightly if one fixates a symbol for too long,” “dissatisfaction if repeatedly a wrong icon was selected,” “electrodes painful after a while,” “high effort needed,” “to realize how diificult it is to focus attention”	“Frustrated if selection wrong”	“Cumbersome, time consuming”
Suggestions for improvements	“Reduce speed of flashing,” “diversify design, so that less tiring,” “no gel,” “quicker text entry method (auto complete function),” “use consistent symbol size”	“Selections could be quicker,” “design,” “design could be optimized,” “improve wearing comfort of electrodes to enable long usage without pain”	“No cap, no wires”	“Might be a help for ALS patients [in the current state]”

**Table 5:** **The average scores of the NASA-Task Load Index (NASA-TLX) and the weighted scores of its subscales for the group of healthy participants that tested the gel-based and dry electrodes**.

**NASA-TLX**		
**Subscale**	**Healthy (Gel)**	**Healthy (Dry)**
Mental demand	8 (±8)	18 (±9)
Physical demand	2 (±2)	0.3 (±0.7)
Temporal demand	7 (±8)	12 (±7)
Performance	10 (±9)	13 (±7)
Effort	9 (±9)	9 (±7)
Frustration	6 (±5)	2 (±2)
Total score	42 (±20)	55 (±15)

*The maximum possible subscale score is 33.33 and the maximum total score 100, with higher score indicating higher workload*.

Effectiveness and comfort were considered the most important aspects of the BCI (each named by 7 participants with the eQUEST2.0 in both experimental groups). While average ratings for the total score of the eQUEST2.0 were above 4 (= quite satisfied), some items scored below this value. In the group that tested the gel-based electrodes, these were speed (3.7) and aesthetic design (3.7) and during testing with the dry electrodes, aesthetic design (3.8) and comfort (3.9). Aesthetic design, comfort and speed were also the items most commented on in the eQUEST2.0. Participants across both groups remarked negatively on the conspicuous design of the electrode cap, but also commented that functionality is more important. Concerning the wearing comfort, participants who tested the gel-based electrodes commented on the electrode cap, two users speculated that wearing it for some time would be annoying. Four users who tested with the dry electrodes also commented on the wearing comfort of the electrodes, which were in direct contact to their skin. One user remarked that the subjective pressure of the dry electrode pins increased over time and another stated that the electrodes were “painful” after wearing them for a while, but he was still “quite satisfied” with the overall wearing comfort. One user commented that he felt a slight pressure of the electrodes, but compared it to a head massage and another user stated that he did feel the electrodes at first, but didn't notice them any longer after wearing them for some time. One user in each group commented that the chin strap was uncomfortable (“a bit too tight,” “pressure on chin”). After testing the gel-based system, a user perceived uncomfortably the ear clip that attached the reference electrode to the user's ear lobe.

Concerning the speed of the system users in both groups commented that it was slightly slow, in particular when comparing the speed to standard computer input methods such as a keyboard or mouse and for complex tasks such as writing a text.

Auto-completion was among the suggestions on how to improve the system named in the usability questionnaire concerning the system design. Participants also suggested abolishing the gel, increasing the wearing comfort of dry electrodes and improving the speed. Most study participants were fascinated that they could give commands using their brain only. Some representative remarks on the positive aspects of the system were: “I would have never thought that something like this was possible,” “positive how easy commands could be executed with the system,” and “exciting to give commands basically just using the brain.” Several comments on the negative experiences concerned the time and high concentration needed for one selection and the difficulties selecting certain symbols: “tiring, because of repetitive flashing,” “frustrating if wrong symbol gets selected,” and “high effort needed.”

On average, the mental workload score as determined with the NASA-TLX was higher for the group that tested the dry electrodes (total score of 55) as compared to the gel-based (42). Substantial differences in the average values for the subscales were observable for the mental demand scale (dry: 18 vs. gel: 8) and the temporal demand (dry: 12 vs. gel: 7).

### End users

Table 6 provides an overview of the main results, in terms of maximum accuracies achieved over all sessions with the prototype, for the end users. Only end users 3 and 5 had sufficient control over the BCI to perform the experimental protocol to test all applications of the prototype. For all other end users, we aimed to maximize the control over the prototype by changing the parameters in an iterative procedure for each user and allow for control over the spelling application. In the following, the results are described in detail for each participant.

**Table 6:** **Overview of results achieved by end users**.

		**Electrodes tested**					
**End user**	**Days of testing**	**Gel**	**Dry**	**Satisfactory control (≥70% accuracy)**	**Max. accuracies achieved**	**Time to complete the experimental protocol**	**Possible obstacles of usage**
01	1	x	✓	No	No classifier could be generated		Insufficient contact of electrode pins
02	1	x	✓	No	No classifier could be generated		Spastic movements of the user
03	1	x	✓	Yes	Spelling: 70% Protocol: 61%	43 min	
04	3	✓	✓	No	Spelling: 60%		Fixation difficulties
05	2	✓	x	Yes	Spelling: 100% Protocol: Error rate 1.5%	23 min	BCI slower compared to his eye tracker
06	3	✓	✓	No	No classifier could be generated		Not possible to combine data from several training runs

*The table lists whether satisfactory control (≥70% accuracy) for the tasks of the experimental protocol or spelling could be achieved and the column “max. accuracies achieved” lists the best performance for completing the experimental protocol or an individual spelling task for each end user. More detailed results for each end user are listed in Section End Users*.

#### End user 1

We could not establish a sufficient signal quality (as assessed by visual inspection of the EEG) with the short electrode pins and a medium size electrode cap. The contact of the dry electrodes on the scalp was not good, caused by the long hair of the user. Testing was discontinued after three unsuccessful attempts to generate classifier weights.

#### End user 2

The dry electrodes could be tightly attached to the user's scalp, but the EEG signal was disturbed by the spastic movements of his left arm (assessed by visual inspection of the EEG). We performed three calibration runs, but the maximum accuracy reached was 5%, thus, no further tests could be performed.

#### End user 3

The electrode cap could be tightly attached to the user's head. After a classifier was created, the participant completed the experimental protocol with a fixed number of 12 sequences per selection. The calibration run estimated a classification accuracy of 30% in the first run and after adjusting the electrodes, a classification accuracy of 34% in the second calibration run. However, the signal quality was judged as good by visual inspection of the EEG. To test whether online spelling was possible using the classifier, we set the number of sequences to 12 (in deviation from the protocol used for healthy participants, who all achieved 100% classification accuracy) to increase the likelihood for successful operation online. During the spelling of 10 letters with the dry electrodes, he reached 70% accuracy and testing was continued with the remaining tasks of the protocol. The participant reached an overall accuracy of 60.6% that resulted in a time to complete the protocol of 43 min. Except for two icons (xbmc in the smart home matrix and the last selection with the web browser), he was able to select all icons within three attempts per icon.

His satisfaction with the device, as rated with the VAS, was 3.8 and he stated that the accuracy should be 100%. The average eQUEST 2.0 score (2.9) indicated that he was more or less satisfied, but the average score of the BCI specific items (reliability, speed, aesthetic design, learnability) was lower (2.2), and he indicated that he was not satisfied at all with the aesthetic design of the electrode cap (rating of 1). He rated ease of use, speed and aesthetic design as most important aspects with the eQUEST 2.0. Although he was not satisfied with the design of the EEG cap (he asked for a method to attach the electrodes that is less conspicuous and does not require a cap), he was satisfied that signals were transmitted wirelessly.

#### End user 4

As can be inferred from Table 7, the best results were achieved on day 3 with the gel-based electrodes (window size adjusted, standard timing parameters). The participant was able to spell 6 out of 10 letters correctly with a fixed number of 14 sequences. None of the other applications were tested due to the low level of control and limited time for testing.

**Table 7:** **BCI performance during testing with end user 4**.

**Session**	**Parameters**	**Estimated classification accuracy**	**Spelling with feedback**
**DAY 1**			
Session 1	Dry electrodes; split screen	1st run: 33% 2nd: 21% 3rd: 17%	N/A
**DAY 2**			
Session 2	Gel-based electrodes, split-screen	31%	N/A
Session 3	Gel-based electrodes; full-screen	39%	0/5 letters (0%) correct
Session 4	Gel-based electrodes ; 200/200 ms; adjusted window size	59%	2/6 letters (33% correct)
Session 5	Gel-based electrodes; 120/80 ms	64%	1/9 letters (11%)
**DAY 3**			
Session 6	Same as in session 5	74%	6/10 letters (60%)

*The “estimated classification accuracy” comprises the values estimated offline for online performance by the system based on the data from the calibration run*.

Although the dry electrodes could be tightly attached to the head of the user on day 1, the EEG signal quality was insufficient for controlling the BCI. We tried to create a classifier three times (as listed in Table 7) with the dry electrodes, but this did not result in accuracies promising for satisfactory online use. As we aimed at maximizing the performance, we tried the gel-based electrodes.

After we had explained the functions of the user interface to the caregiver and taught him how to set up the electrode cap, the caregiver felt confident that he could operate the interface with a little practice and could include the set-up of the BCI in his daily routine. He stated that it contained all the relevant functions, was intuitive and remarked that it was “easy to understand and clearly laid-out.” He had no suggestions on how to improve the system. The patient was generally satisfied with the GUI, but stated that it might be “over featured,” meaning that the high functionality of the program could possibly distract the user. He suggested to display only the most important options and to display the other settings only in an expert mode.

#### End user 5

The participant did not like the pressure of the dry electrodes, therefore we refrained from testing this electrode type.

In the first session, the calibration run was successful and he achieved 100% classification accuracy with an estimated number of 3 sequences with the gel-based electrodes. He needed 23 min (65 selections) to complete the experimental protocol. The overall accuracy was 64.6% with 33.9% of selections suppressed and only one erroneously chosen selection (1.5%). He was able to select all but three icons, with a maximum of three attempts per icon.

He rated overall satisfaction with the device as 4.5 on the VAS. The total eQUEST2.0 score (3) indicated that he was more or less satisfied with the prototype. The average score of the added items was a bit lower (2.5), and he was least satisfied with the speed of the system (rating of 1). He compared the BCI prototype to his eye tracking system that allows him to make selections more rapidly and which is less complex. On the other hand, he stated that it was “pretty easy” to set up the electrode system and adjust the cap and could imagine that other patients could benefit from the technology. He criticized the material of the electrode cap since he started to sweat after wearing it for some time.

In the second session, the set up was performed by the participant's wife and he was lying in his bed. The first calibration run was successful (100% with 5 sequences) and he copied a five letter word correctly. Afterwards he tested the system (mainly the web browser) for about 1.5 h and both he and his wife provided feedback. With a VAS ranging from 0 to 10, the end user's wife indicated that the setup of the electrode cap and the software was easy (nine). She stated that starting the software was easy and she could incorporate the setup into her daily routine. To setup the system on her own, she estimated that she would need two more practice sessions with guidance.

The patient suggested implementing a pause of several seconds after a web page has been loaded before selections could be made to provide the user with sufficient time to find the desired command in the control matrix. He stated that he was not satisfied with the amount of wrong selections, which occurred particularly often when he was not paying attention to the control matrix.

#### End user 6

Although we tested several settings (dry and gel-based electrodes, different screens and cap sizes) during the first day, attempts to classify the data were unsuccessful. The software of the prototype did not allow for combining data from several calibration runs. To test whether more training data could lead to a performance improvement, we used the BCI2000 software on the second day of testing and performed 7 runs of spelling. The combination of the first two runs yielded an estimated classification accuracy of 20%. Afterwards we darkened the room and performed an additional run, for which the estimated classification performance was 80%. The participant performed 4 more runs, in which she was asked to spell five letters. In each run feedback about the chosen letters was provided to the participant. In a stepwise procedure, each run was added to the runs used to train the classifier. With this iterative process online classification accuracy increased from 40% in run 4 to 60% in runs 5 and 6 and finally to 80% in run 7. After the seventh run, the participant was exhausted and no further runs were performed.

On the third day, we tested the prototype with the dry contact electrodes again. The calibration matrix was displayed on her large screen TV. Within two calibration runs, the calibration was not successful. Therefore, we stopped the BCI session and asked her and the caregiver to demonstrate the eye tracking system. The dwell time was set to 1.5 s. She could not select all symbols equally well with the system. The error rate was particularly high for neighboring symbols and she needed several attempts to selects icons displayed on the outer left side of the screen. Depending on which symbols were chosen, her accuracies ranged between 50 and 100%. She indicated that she could use the eye tracker for about 1 h, before she needed a longer break. We tested whether it was possible to improve classification accuracies by increasing the dwell time to 2.5 s, but she was unable to focus on one particular item for this amount of time.

At the end of testing, she estimated that she could wear the gel-based electrode cap for about 2 h and the dry electrodes for about 1 h. She stated that her eye tracking system worked better compared to the BCI system. Nevertheless, she indicated that she would like to continue testing BCIs, but would prefer auditory over visual systems.

Her caregiver had observed the setup on 2 days of testing and felt confident that she could include the set-up of the BCI in her daily routine.

### General remarks about testing with end users during supervised sessions

The experimenters noted that the wireless signal transmission facilitated the set-up in the home environments as compared to previously used tethered electrode systems. Although the application of gel was not required for the dry electrodes, a considerable longer time was needed by the experimenters to adjust the cap and establish a good signal quality (not quantitatively assessed). The signal was more easily disturbed by movements of the user, movements of persons nearby or electrical noise in the environment during testing with the dry electrodes as compared to the gel-based electrodes. A possible explanation is a higher impedance of the dry electrodes.

## Discussion

The BCI prototype implemented within the project *Backhome* allows for muscle-independent control over various digital applications ranging from spelling to web browsing. The evaluation with healthy participants demonstrated that it could be operated with satisfactory accuracies with gel-based and dry contact electrodes. All participants could spell a given word correctly independent of the type of electrodes. During testing across all applications, the percentage of false selections was lower for the gel-based as compared to the dry contact electrodes. While during testing with the dry electrodes, a selection was made after a fixed number of 10 flash sequences, a dynamic stopping method was activated during testing with the gel-based electrodes. It served its purpose of minimizing the number of false selections and substantially speeding up selections for some participants. However, a large number of selections was suppressed (no selection after 10 sequences), in particular for the smaller matrices. Previous studies demonstrated that achieved accuracies do not depend on matrix size (Sellers et al., 2006) nor on the layout of the matrix (Nam et al., 2009). These studies differed from our study in that calibration and testing matrices were equal. Therefore, future studies could systematically investigate to what degree performance is affected by the difference in matrix size of the calibration and control matrix. If there was a dependence as hinted in our explorative analysis (Figure 7), the icons in all control matrices should be similar in size to the ones in the calibration matrix to prevent a high number of suppressed selections.

### Dry electrodes

For healthy users, the satisfaction ratings did not differ substantially for dry and gel-based electrodes. However, comfort ratings as expressed with the eQUEST were on average slightly lower (3.9) for the dry electrodes as compared to the gel-based electrodes (4.4). In the group that tested the dry contact electrodes, comments on the wearing comfort varied substantially between users. While the majority of users did not complain about the wearing comfort, several users negatively commented on the pressure exerted by the dry electrodes and one described them as painful. The design of the electrode was a topic that was addressed by several users, it was described by both user groups alike as conspicuous (gel and dry).

The workload as assessed with the NASA-TLX was estimated higher (on average) by the group that tested the dry electrodes as compared to the group that tested the gel-based electrodes. Substantial differences were found in the subscales mental and temporal demands.

This suggests that the dynamic stopping method that was used in combination with the gel-based electrodes helped to reduce the workload. For the dry electrodes, participants needed in general longer for each selection since always 10 sequences were needed, whereas for the gel-based system the number was determined dynamically. Consequently, participants had to focus attention for a longer time in the group that tested the dry electrodes, increasing the mental demands.

The two available pin sizes for the dry electrodes offered a level of adjustability that was sufficient for most, but did not allow optimal adjustment for all users. However, the testing with healthy participants demonstrated that satisfactory accuracies could be achieved with the dry contact electrodes, thus they expand the range of sensors to choose from. The achieved accuracies of 87% with a fixed number of 10 sequences for an average of 55 selections are in the range previously reported by Pinegger et al. (2016), but on average higher. This could be explained by individual differences (due to small sample sizes of both studies and high inter-individual differences) or the fact that a different amplifier and electrode cables were used by Pinegger et al. (2016). In our study, ribbon cables were used with a fixed, individual length for each electrode and connected to the g.Nautilus amp that was attached to the back of the cap, whereas a standard g.USBamp and cables were used by Pinegger et al. (2016). Reduced time for EEG recordings with dry electrodes, because no application of gel is required, is often named a possible advantage of dry electrodes (e.g., Zander et al., 2011; Lopez-Gordo et al., 2014). In line with the experience reported by Pinegger et al. (2016), we can state that it was time consuming to find a tradeoff between wearing comfort of the cap (pressure of pins against the skin not too high) and good signal quality and that the system was easily disturbed by movement artifacts. Although not quantitatively assessed, and unlike expected, the preparation/time needed to setup the dry electrodes until a reliable signal was reached was equally long or even longer as compared to the gel-based system judging from our experience with healthy participants and end users.

With improvements regarding the wearing comfort and reliability of the signal, dry sensors may become an option for use in daily life. Spring-loaded metal pins (Lo et al., 2016) or soft and conductive material (Lin et al., 2011; Yu et al., 2014) could improve the wearing comfort. Enabling a good wearing comfort of the electrodes is of particular importance for end users with severe paralysis. End user 5 refrained from testing the dry electrodes that caused a feeling of uneasiness/discomfort, because after his spinal cord injury the head remained the only body part with intact sensation/was particularly sensitive and he did not want to take any risks. Another factor that might be problematic during real-life use in less-controlled environments is that dry electrodes usually have higher impedance values as compared to gel-based electrodes, and they might be more susceptible to interference caused by movements or ambient electrostatic charges (Chi and Cauwenberghs, 2010; Chi et al., 2010; Guger et al., 2012; Lopez-Gordo et al., 2014).

### Toward better, less conspicuous electrodes

As proposed in this and previous studies, small head-mounted amplifiers and wireless signal transmission can facilitate EEG recordings outside the lab (Debener et al., 2012; De Vos et al., 2014). Electrodes that are fixed in a cap made of flexible material are currently the prevailing method for the recording of high quality EEG signals. In the future, further efforts are necessary to design non-invasive electrode systems that are inconspicuous and can be attached for several hours without causing discomfort. Individualized ear-pieces with electrodes placed in the ear (on the concha and in the outer ear canal) of users is a less noticeable option under investigation (Looney et al., 2011, 2012; Bleichner et al., 2015). Bleichner et al. (2015) could demonstrate that the P300 could be reliably detected with an electrode placed on the upper concha during a BCI spelling task. An even less obtrusive recording method was recently suggested by Debener et al. (2015). They developed miniaturized, electrodes printed on a flexible, c-shaped sheet that allows for recordings of EEG signals from the non-hair-bearing area around the ears. Reliable P300 signals could be recorded during an auditory oddball task even after participants wore the electrodes for more than 6 h.

### Toward home use of BCI

The performance of the BCI varied substantially between the potential end users. While end user 5 achieved accuracies in the range of healthy controls and could control all applications, end users 4 and 6 did not even achieve satisfactory accuracies with the spelling application and for end users 1 and 2 no sufficient EEG signal quality could be obtained to control the BCI (with a medium size cap and dry electrodes with short pins). Because of the small number of participants, we cannot systematically evaluate the dependence on the level of impairment. However, end-users 4 and 6 were the most severely paralyzed. Both could not control an eye tracking system, probably due to their limited control over eye movements. This might be one of the reasons why they were unable to control the BCI. Although both indicated that they were able to see all elements of the matrix properly.

Brunner et al. (2010) demonstrated in a study with healthy participants that performance with a P300 matrix speller depends on gaze direction. Participants were asked to fixate a cross in the center of the matrix during a spelling task. Their performance declined with increasing distance of the target letters from the fixation point (i.e., the more peripheral the target was displayed). For end user 6, we could confirm in our study using her eye tracker that the participant had difficulties fixating her gaze on a particular point for more than a few seconds.

For the most severely paralyzed users, with no or only little control over eye movements and/or visual impairments, BCIs that do not rely on eye gaze were proposed (for a review see Riccio et al., 2012). In recent years several paradigms were proposed that depend on auditory or tactile stimulation (Höhne et al., 2011; Schreuder et al., 2011; Kaufmann et al., 2013a; Hill et al., 2014; Käthner et al., 2015a; Kleih et al., 2015; Simon et al., 2015; Halder et al., 2016; Herweg et al., 2016). These paradigms could prove valuable for persons with most severe paralyses. Recently, Halder et al. (2016) and Herweg et al. (2016) demonstrated that potential end users could gain control over an auditory spelling application with training and control a tactile virtual wheelchair. Because these systems are still in the proof of concept stage, we did not implement them in the present prototype, but they should be considered for future systems.

The caregiver of end user 5 conducted the setup of the EEG system and started the BCI by herself, rated it as easy and felt confident that she could incorporate it in her daily routine. The caregivers of end user 4 and 6, who had observed the setup, also felt confident that they could perform the setup as part of their daily routine. Feedback from them indicates that the design of the software is intuitive. However, we did not evaluate in this study, whether they could perform the setup without expert supervision. Miralles et al. (2015b) presented the results from an independent home use phase of the system with people living with acquired brain injury during which caregivers did the set-up without direct expert supervision. The caregivers were able to do the set-up at home but occasionally encountered technical problems that made it challenging for them to support the EEG measurements as part of their daily routine and was only possible with high commitment and patience. In exemplary patients independent home use of BCI has already been demonstrated (Sellers et al., 2010; Holz et al., 2015a,b).

### Technical improvements

Important improvements for BCI systems that are intended to be used as assistive technology should include the option to train the classifier with more data (e.g., by combining several calibration runs). This is particularly important for dynamic stopping methods since their performances improve with larger training set sizes (Schreuder et al., 2013). The implemented dynamic stopping method in our prototype served the purpose of minimizing false positive selections. However, applications could be further optimized to prevent involuntary selections if the user is not paying attention to the screen. For instance, Pinegger et al. (2015) implemented a method that takes into account additional features from the frequency domain to accurately detect the user's control state. To improve the speed of the system for the spelling application, auto-completion algorithms could be implemented (Höhne et al., 2011; Kaufmann et al., 2012).

### Lessons learnt from the evaluation with end users and consequences for future BCI research

Apart from technical improvements that can be derived from the evaluation with end users, there are other (more fundamental) questions that can be raised following the evaluation of the prototype. We aimed to develop a BCIs with high functionality that is easy to use for non-experts (patients and caregivers) and can be integrated into their daily life.

Although there is generally a high interest by potential end users in a wide range of applications (e.g., control over a computer, a wheelchair and smart home control; Huggins et al., 2011) taking into account the individual user's needs is highly important.

While healthy participants were able to gain control over the BCI prototype, the end users with the most severe paralyses (end users 4 and 6), who should benefit the most from the system, were unable to achieve control over the BCI despite various efforts to adjust parameters to their needs during the measurements. Furthermore, end user 5, who achieved very high accuracies was not very satisfied with the BCI in general and not satisfied at all with the speed of the system. His achieved accuracies were in the range that survey participants with ALS and spinal cord injury named as acceptable for BCIs (>90% accuracy; Huggins et al., 2011, 2015). But these surveys also demonstrated that the desired speed (>20 characters/min) cannot yet be fulfilled by most BCI systems. Even for healthy users the speed with the *Backhome* system was far below this value (gel: 2.8 selections/min; 1.8 correct selections/min, dry: 1.9 selections/min; 1.6 correct selections/min). In previous studies substantially higher selection rates were reported for ERP-BCIs in studies with healthy participants. For instance, Lenhardt et al. (2008) reported selection rates of up to 4.4 correct selections/min and Käthner et al. (2015b) average selection rates of up to 9.9 correct selections/min. However, in both studies users spelled only a restricted amount of letters (22 and 17 respectively) and it remains to be demonstrated that such selection rates could also be achieved during sustained ERP-BCI tasks.

End user 5 compared the BCI to his eye tracker, which was much faster and had an even lower rate of undesired selections. Rather than developing a system with high functionality that targets a wide range of users, it might me more important to develop systems according to individual users' needs specifically and/or provide functionality that cannot be provided by standard assistive technology. For instance, in the study by Huggins et al. (2015), persons with spinal cord injury emphasized the importance of functions that may not be otherwise available to them (e.g., control of a robot arm). None of the potential end users in the evaluation studies reviewed by Kübler et al. (2014, Zickler et al., 2011; Riccio et al., 2015) could imagine using the BCI for communication in daily life. Potential end users were much more tolerant regarding errors of BCI applications aimed at entertainment. That a match between the end users' needs and the technology can result in satisfactory BCI use in daily life was recently demonstrated (Holz et al., 2015a,b). For independent home use, two end users in the locked-in state were provided with the Brain Painting application for artistic expression. Although the estimated accuracies were only in the range of 70-90% and there were technical challenges over the course of several months of testing (15 and 22 months respectively), both were highly satisfied with the BCI (Holz et al., 2015b). These findings underline the importance of stringently following the user-centered design approach to achieve a match between the users' needs and the BCI technology (Kübler et al., 2014). For the most severely paralyzed patients, emergency communication is likely to be the BCI task with the highest priority if all other assistive technology fails and providing BCI based communication for persons without motor control remains the most important challenge (Kübler and Birbaumer, 2008; Huggins et al., 2015). To ease the transition to a BCI as sole input method to assistive technology once control over all motor activity is lost, hybrid BCIs were proposed (Millán et al., 2010). These hybrid BCIs combine standard assistive technology with a BCI so that users can switch between different input methods, e.g., eye tracking, a joystick and a BCI (Pfurtscheller et al., 2010; Amiri et al., 2013). Combining different input methods and adjusting BCI parameters for individual users to optimize BCI performance is a time consuming service that cannot be provided by researchers in the long run and needs to be undertaken by providers of assistive technology.

## Concluding remarks

BCIs are intended as muscle-independent communication aids for persons with severe paralysis, yet studies with target users are scarce and most studies are conducted in controlled laboratory environments. It is a crucial yet challenging step from BCIs requiring expert knowledge to easy-to-use systems. Our development efforts within the *Backhome* framework resulted in a multi-functional BCI that provided applications for communication, web access, entertainment, artistic expression and environmental control.

The presented evaluation study can help to pave the way for BCIs intended as assistive technology for persons with severe paralysis. The herein presented results with healthy participants and potential end users in their caregiving environments revealed advantages and disadvantages of both the hardware and the software. The user-centered design approach needs to be followed more strictly to provide a match between end users' needs and BCI technology (Kübler et al., 2014).

## Author contributions

IK conducted the study, analyzed the data and drafted the manuscript. SH and AK helped to draft the manuscript. CH, CG, AE, SD, EV, XR, and MS designed and implemented the employed soft- and hardware. IK, JD, SM, and AK conceived of the study. All authors contributed to writing the manuscript.

### Conflict of interest statement

CG and CH are full-time employees of g.tec medical engineering GmbH, Guger Technologies OG, and CG is its co-CEO and owner. The other authors declare that the research was conducted in the absence of any commercial or financial relationships that could be construed as a potential conflict of interest.

## Footnotes

^1^http://cordis.europa.eu/project/rcn/101842_en.html
